# Flipped learning as an educational model in a cardiology residency program

**DOI:** 10.1186/s12909-023-04439-2

**Published:** 2023-07-17

**Authors:** Carlos R Sierra-Fernández, Huipe-Dimas Alejandra, Sergio A Trevethan-Cravioto, Francisco J Azar-Manzur, López-Meneses Mauricio, Luis R Garnica-Geronimo

**Affiliations:** Teaching Department, National Institute of Cardiology, Mexico City, Mexico

**Keywords:** Flipped learning, Medical Education, E-learning, Blended learning, Cardiology, Medical residency

## Abstract

**Introduction:**

Flipped learning (FL) is a model which allows students to adjust their study rhythm by taking advantage of class time to apply their knowledge. Although FL meets many of the needs of the traditional lecture-based model and the exclusively virtual model, its effectiveness in medical residency programs has not been thoroughly studied. Our study evaluates the efficacy of an FL model in a cardiology residency program based on the satisfaction and academic performance of the participants.

**Method:**

A prospective, observational, and descriptive study was conducted at Ignacio Chávez National Institute of Cardiology in Mexico City to evaluate the satisfaction and effectiveness of an FL model for acquiring competencies in electrocardiographic diagnosis among thirty-one first-year cardiology residents. The Advanced Electrocardiography Workshop and a virtual classroom were designed for the intervention. Four teachers taught the workshop and video classes, and ten medical specialists from the participants’ areas of work performed evaluations of practical skills before and after the intervention.

**Results:**

75% of the participants rated the intervention as very favorable compared to the traditional and virtual models. The main advantages offered by the FL model were identified. An improvement of high statistical significance was observed in participants’ academic performance after the intervention (*P value < 0.001*).

**Conclusion:**

The FL model has a high degree of acceptance among participants and teachers. Our study shows an improvement in academic performance after the intervention. Considering that the FL model is flexible and reproducible in other areas of medicine, it represents an excellent educational alternative that meets current needs.

## Introduction

Socratic questioning serves as the ultimate form of argumentative dialectic, with the teacher assuming a paternalistic role in guiding the disciple through discussions on a topic that is familiar to both but mastered by the teacher. This principle was later embraced by Plato in the establishment of the Academy. In The Banquet, Diotima compares the philosopher to a midwife, stating that “the soul is pregnant and wants to give birth,“ drawing an analogy between knowledge and childbirth. In contemporary idealism, the essence of the flipped learning (FL) model lies in the Socratic dialectic, where the student possesses knowledge of the subject and the teacher acts as a guide in facilitating knowledge discovery. Rather than merely transmitting knowledge, the focus is on teaching its correct application and reasoning [1].

The traditional lecture-based model (LBM) directly provides the student with knowledge during class time, while time outside the classroom is dedicated to reinforcing, practicing, and reflecting on this knowledge. One of the negative points of this model has been the mismatch between the student’s learning style and the teacher’s teaching style, which was the main problem that led to the proposal of alternative educational strategies during the second half of the 20th century and the beginning of the 21st century [2–5]. Flipped learning (FL) proposes that educational content (core content) should be reviewed through physical means (books and journals) or electronic means (e-books, prerecorded classes, and podcasts) outside of class time, which allows students to adjust their own learning pace. In contrast, the class time should be allocated to applying this knowledge, its discussion, and reflection, thus reinforcing knowledge acquisition through active learning.

The success of this model and the ease of its reproducibility catapulted its use in various educational contexts; medical education was no exception. Multiple studies have evaluated FL’s effectiveness in acquiring medical knowledge in undergraduate and graduate medical education. Studies that compared knowledge and skills acquisition through the FL model *versus* LBM showed heterogeneous results; although some found a significant difference [6–10], others found a marginal difference [11, 12]. In contrast, satisfaction was higher among students exposed to FL, highlighting their ease in adjusting their learning pace and the reinforcement of knowledge in the classroom, reflected in increased motivation for learning [6–12]. Although these studies have been carried out predominantly in undergraduate medicine programs, those carried out in residency programs have highlighted that the main limitation of FL is the resident’s limited availability to study the core content outside of class time, considering that their primary learning occurs in the hospital and that the workload limits their time outside of it [13].

Cardiology teaching has recently integrated new technological tools for acquiring and reinforcing knowledge [14]. However, research in cardiovascular medical education has been limited, reflected in the scarce implementation of new educational models [15]. Although the use of FL has been extended to teaching other branches of medicine, its implementation in cardiology training has been particularly weak [15].

FL is a tool applicable to the hospital environment that meets the needs posed by the limitations of LBM in medical education. The objective of this study was to evaluate the satisfaction rates and effectiveness of an FL model among first-year cardiology residents.

## Method

### Design

A prospective, observational and descriptive study was carried out to evaluate the satisfaction and effectiveness of a flipped learning model among first-year cardiology residents for the acquisition of electrocardiography competencies. It was carried out in the facilities and with the participation of the academic staff of Ignacio Chávez National Institute of Cardiology of Mexico City. Prior to the start of the intervention, all participants were evaluated through an examination of theoretical knowledge in electrocardiography. In turn, medical specialists from the participants’ areas of work individually assessed the performance of 5 core competencies in electrocardiography (Table 1). For the intervention, a virtual classroom was designed through which participants could access asynchronous virtual classes, which could be taken at any time, allowing each participant to individualize their study rhythm. In parallel, the Advanced Electrocardiography Workshop (AEW) was implemented, a program of face-to-face academic activities designed to apply the knowledge acquired through the virtual platform. In this workshop, expert electrocardiography teachers guided the participants through the electrocardiogram (ECG) analysis process through clinical case discussions and problem-based learning.

**Table 1:** Core competencies evaluated by the medical specialists from the participants’ areas of work

**Competence 1.** Definitive diagnosis
**Competence 2.** Differential diagnosis
**Competence 3.** Electrometry
**Competence 4.** Identification of the electrocardiographic technique
**Competence 5.** Integration of data in decision-making

To evaluate academic progress, at the end of the intervention, an examination of theoretical knowledge and an evaluation by the medical specialists regarding the acquisition of the five competencies was carried out individually. Participants and teachers were surveyed to determine their satisfaction with the FL model.

### Study participants

A total of 35 first-year cardiology residents were invited, all of whom were medical graduates who had completed training in internal medicine as a prerequisite and were enrolled in the first year of cardiology residency during the third quarter of 2021. We had the support of 4 teachers who conducted the AEW and the asynchronous video classes; all of them are cardiologists with extensive experience in electrocardiography. They monitored the residents during the mentoring, training, and feedback process. Additionally, ten medical specialists from the participants’ areas of work objectively carried out personalized evaluations of the five core competencies in electrocardiography before and after the intervention.

### Evaluation and analysis

The first theoretical knowledge examination was carried out a week before the intervention, and the second was carried out on the last day of the AEW. In both cases, the participants had a time limit of 1 h and 30 min to complete the examination. The authors developed both examinations. Each one included 20 clinical cases that evaluated six electrocardiography topics. Examinations were graded on a scale of 0-100; the items were related to the systematic and deductive analysis of ECGs. The exam topics were used to design the modules of the flipped learning intervention. Results data were analyzed with SPSS Statistics, version 27.0 (IBM Corp., Armonk, NY; USA). We confirmed the normal distribution of the results obtained from the pre and post intervention examinations using a Shapiro-Wilk test (W = 0.963, P = 0.313). To determine the statistical significance of the results we employed a t-test. *Canvas* (Instructure Inc., Salt Lake City, USA) was used for the development of the virtual classroom. It is worth noting that the platform is open access, allowing both users and developers to freely access and utilize its features, it also allows the establishment of two-way communication channels. The academic content was divided into six modules based on the topics evaluated in the first exam (Table 2). In total, 19 asynchronous video classes were loaded with an average duration of 20 min each.

**Table 2:** Academic content used in the virtual classroom and Advance Electrocardiography Workshop (AEW).

Module	Main topic	Video class	Classroom activities
**Module 1**	**Electrocardiography overview**	1: Electrophysiological bases of the ECG 2: Normal ECG	a) Gamification activities b) Clinical case related to the topic c) Deductive ECG analysis
**Module 2**	**Cardiac enlargement**	3: Atrial enlargement ECG 4: Ventricular enlargement ECG	
**Module 3**	**Conduction disorders**	5: Branch blocks 6: Fascicular blocks 7: Atrioventricular blocks 8: Sinoatrial blocks	
**Module 4**	**Ischemic heart disease**	9: ECG in ischemic heart disease 10: ECG in acute myocardial infarction	
**Module 5**	**Arrhythmias overview**	11: Mechanisms of arrhythmias 12: Supraventricular tachycardia 13: Ventricular tachycardia 14: Atrial fibrillation and flutter	
**Module 6**	**Arrhythmic syndromes**	15: Preexcitation syndromes 16: Early repolarization 17: Long QT 18: Brugada syndrome 19: Channelopathies	

*ECG Electrocardiogram*

The workshop included 13 academic sessions distributed over eight weeks. As the first activity, the sessions included introductory gamification activities assessable through Kahoot! (Kahoot Inc., Norway), where the knowledge acquired through the video classes was applied. The first 15 min of each session were allocated to this activity. The remaining time (65 min) was used to perform ECG analyses based on the presentation of clinical cases according to the corresponding topic. Each academic session lasted 80 min.

A rubric was created wherewith medical specialists personally evaluated the five core competencies in electrocardiography before and after the intervention; based on their judgement, the physicians indicated whether the participant had that competency.

The degree of satisfaction of both the participants and teachers was evaluated through a comparative analysis of the LBM, the VM, and the FL model. The teachers were asked about their degree of satisfaction with the FL model, with five responses: *very satisfied, satisfied, neutral, dissatisfied, and very dissatisfied.* A 3-part survey was applied to participants in which two questions asked them to express their degree of satisfaction with the intervention compared to the LBM and VM. There were four response options for these questions: *very favorable, favorable, neutral, and unfavorable*. The third part of the survey asked the participants to select the FL model characteristic they considered the main advantage over the LBM. Four options were provided: *less theoretical content in face-to-face classes, asynchrony in the review of theoretical content, face-to-face interaction after reviewing theoretical content, and having the support of the theoretical content*. The intervention lasted eight weeks.

## Results

Thirty-one residents voluntarily agreed to participate in the study, of whom 25 were male (80.5%) and six were female (19.5%). Throughout the intervention, good involvement was obtained among participants and teachers, reflected in the number of visits to the platform and the satisfaction results. Five thousand nine hundred seventy-two visits were recorded in a virtual classroom over the eight weeks, an average of 192.6 visits per student.

### Satisfaction

Three of the four teachers (75%) expressed being very satisfied with the FL model, while one of them (25%) described being satisfied.

Twenty-three of the thirty-one participants (74.1%) rated the intervention as very favorable compared to the LBM, four as favorable (12.9%) and four as neutral (12.9%). Compared with the VM, twenty-eight participants (90.3%) rated it as very favorable and four (9.7%) as favorable.

**Fig. 1:**
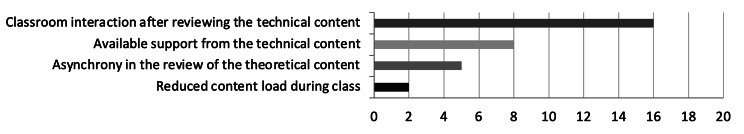
Voting results for the main advantages of the flipped learning model

Regarding the primary benefit of the FL model, the participants were presented with four choices and opted for the one they deemed to be the most significant. Out of the 31 participants, 16 individuals (51.6%) voted in favor of *Classroom interaction after reviewing the technical content*; 8 participants (25.8%) chose *Available support from the technical content*, while 5 participants (16.1%) elected *Asynchrony in the review of the theoretical content*. Finally, 2 participants (6.4%) selected *Reduced content load during class* as the FL’s primary benefit. (Fig. 1).

Gamification activities were not considered during the analysis of academic progress data but instead served as a self-assessment method for the participants.

### Academic progress

**Fig. 2:**
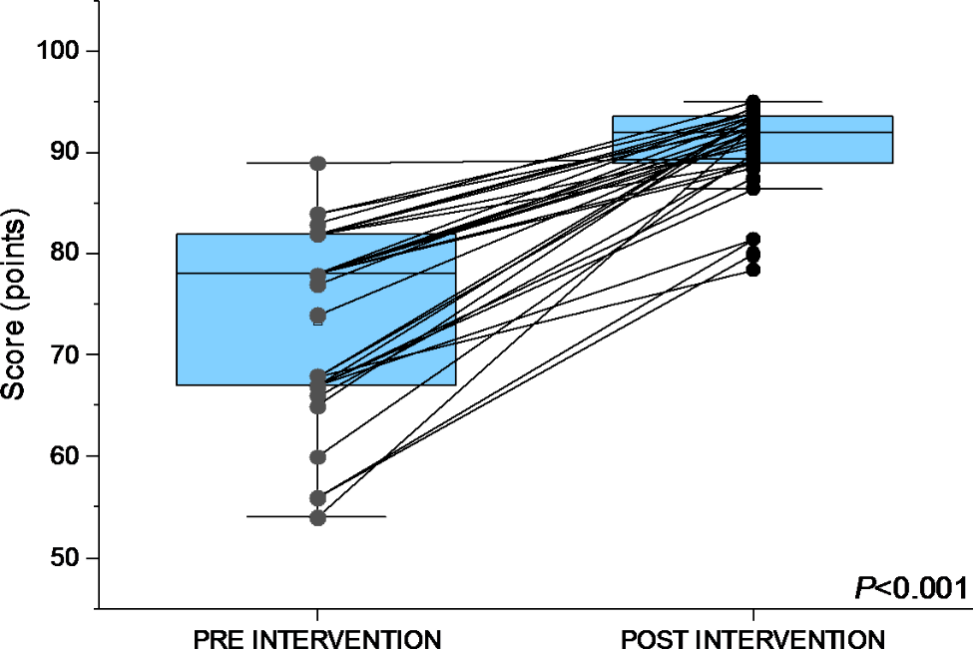
Scores obtained by the participants in the theoretical examination

The average score on the theoretical examination applied to the 31 participants before the intervention was 73.4 (95% CI, SD ± 9.36) and the average score on the test after the intervention was 90.1 (95% CI, SD ± 4.4), an increase of 21% (*P value < 0.001*) showing thus a statistically significant improvement (Fig. 2).

Based on the criteria of the medical specialists, satisfactory results were obtained regarding the progress of the participants in the five core competencies in electrocardiography. Of the thirty-one participants *Competence 1* was already present in eighteen participants (58%) at the end of the intervention it was present in twenty-seven (87%); *Competence 2* was already present in twenty-nine (93.5%), *Competence 4* in twenty-six (83.8%) and *Competence 5* in twenty-seven (87%), at the end of the intervention the 100% of participants acquired the competencies.

**Fig. 3:**
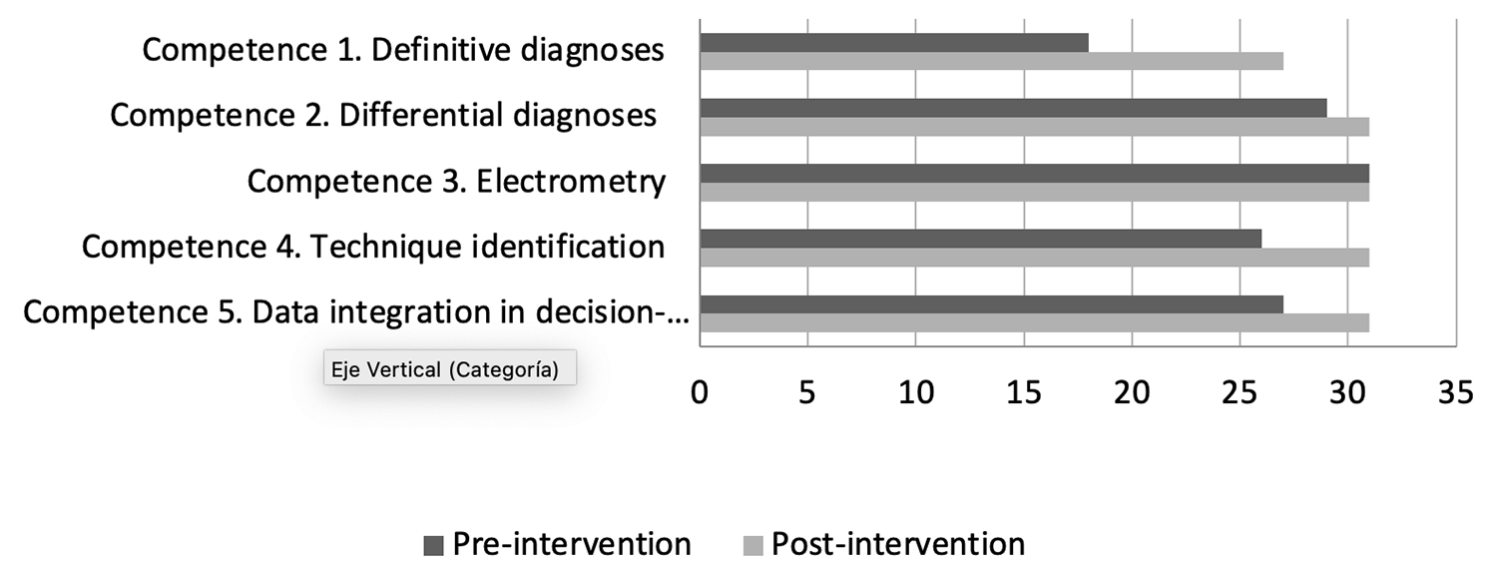
Participants’ results in the five core competencies, based on medical specialists’ evaluations

Evaluation of Electrometry (Competence 3) did not differ before and after the intervention, as 31 participants had already acquired this competency (Fig. 3).

## Discussion

Our study is one of the few conducted on FL in a medical residency program, and the first carried out in a cardiology residency program, however, some studies have previously evaluated the efficacy of FL in the ECG-interpretation abilities acquisition, all of them in undergraduate medical education and nursing training programs [10, 16, 17]. The results for the aspects evaluated during this study were positive.

In the authors’ perception, the teachers’ and participants’ motivation was significantly higher for the FL model than for the LBM. Participants were significantly involved in the process, and participation was markedly more heightened during the practical sessions; this was directly reflected in the satisfaction results obtained after the intervention, indicating that teachers and participants preferred FL over the traditional and virtual models. This finding agrees with what has been reported by other FL studies carried out in medical education, where the trend that favors it is invariably predominant [6–12]. The acceptance of VM among the medical community has been heterogeneous [18]. Some studies have identified its main disadvantages compared to the LBM; the most notable are the little interaction and the ease of losing concentration in a non-school environment [19].

Using a gamification tool (Kahoot!) made the case analysis sessions dynamic and attractive for the participants while providing a practical and attractive self-assessment system.

Numerous studies have conducted assessments on the effectiveness and extensive adoption of gamification tools within medical residency programs, resulting in a positive impact on students’ motivation and commitment [20, 21]. This type of educational assistant requires greater dissemination among health sciences teachers. In the authors’ opinion, although the pandemic increased the use of new educational methods, this was not reflected in the use of gamification tools.

Unlike the satisfaction results, the acquisition of knowledge and skills in the FL model has been challenging to reproduce, generating heterogeneous results among the multiple studies that have applied this model to medical education. In this study, knowledge acquisition was significantly improved compared to the results obtained before the intervention (*P value < 0.001*); the evaluations support this finding by a third party (medical specialists) and by the authors (theoretical knowledge exam). We consider the success of an FL model not only depends on reversing the traditional teaching and learning modality but also on aspects related to logistics, the quality of teaching, and the commitment of the student and teacher. Since a medical residency program hinders synchronous learning models, logistics plays a fundamental role in designing a structured program that allows participants to allocate time for reviewing the subject outside of the classroom and for applying knowledge during class, which is essential for the success of the intervention. A failure in this aspect could limit the reproducibility of the model. Some studies have highlighted that the effectiveness and students’ engagement with the FL model in medical education are determined by a complex interaction of factors. These factors include variations in teaching styles, individual learning preferences, the level of guidance during self-study, and the motivation of both teachers and students. It is important to note that most of these observations primarily derive from studies conducted in undergraduate medical education [22, 23]. More studies are needed to determine what other factors influence the acquisition of skills and knowledge in FL models applied to medical residency programs.

In terms of cost-effectiveness, we used an open access platform (*Canvas*) for our FL model enabling us to provide the intervention to our participants free of costs. Considering the wide range of open access options available on the internet, the implementation of FL emerges as a convenient and cost-effective educational alternative.

One of the benefits of using a flipped learning model in medical education is that the time invested by the instructors in creating recorded lectures is equivalent to the time spent in delivering traditional in-person lectures. However, with recorded lectures, the instructors have the advantage of having the content available on the platform for students to access at any time and as many times as necessary. This can be particularly beneficial for students who may need more time to review the material, or for those who want to review the lecture before an exam or clinical rotation. While the upfront time investment may be greater for instructors in creating recorded lectures, the potential long-term benefits for students can make it a worthwhile investment. Additionally, the flexibility of the flipped learning model can allow instructors to spend more time engaging with students during in-person sessions, further enhancing the educational experience. The benefits of a flipped learning model extend beyond the current course and can be utilized for future classes as well. Once the recorded lectures are created, they can be reused for future classes, reducing the time and effort required for instructors to prepare lectures.

## Conclusion

FL is a reproducible educational strategy applicable to teaching in medical residency programs, with high satisfaction levels reported by students, however, its success in knowledge acquisition depends on multiple factors. The FL model is particularly attractive and viable today, as it provides physicians-in-training with a flexible and personalized model that overcomes the limitations of traditional and virtual learning. The intervention confirmed that having undergone training based on the FL model was helpful for acquiring skills and knowledge in electrocardiography among cardiology residents. Although the LBM continues to dominate medical education programs, it is imperative to continue implementing new models that meet current needs and integrate technological resources that facilitate the learning experience. When well applied, FL is a successful alternative for teachers and students.

### Limitations of this study

Our study has some limitations that should be considered. First, due to the practical challenges, particularly when involving medical residents, our study did not include a control group. Second, our study did not assess the long-term retention of ECG interpretation skills. Therefore, it is unclear whether the improvements observed in our study are sustainable over time. Future studies should consider including a control group and evaluating the long-term retention of ECG interpretation skills to better understand the efficacy of the flipped learning model in medical education.

## Data Availability

The datasets generated and/or analysed during the current study are available in the next persistent web link: https://drive.google.com/drive/folders/1IiTIUzVsEn1eCuly4UeH5pk2aIqmxNSO?usp=share_link.

## Abbreviations

AEW: Advanced Electrocardiography Workshop
FL: Flipped learning
LBM: Traditional lecture-based model
VM: Exclusively virtual model.
